# Accessory Genome Dynamics and Structural Variation of *Shigella* from Persistent Infections

**DOI:** 10.1128/mBio.00254-21

**Published:** 2021-04-27

**Authors:** Rebecca J. Bengtsson, Timothy J. Dallman, Hester Allen, P. Malaka De Silva, George Stenhouse, Caisey V. Pulford, Rebecca J. Bennett, Claire Jenkins, Kate S. Baker

**Affiliations:** a Clinical Infection, Microbiology and Immunity, Institute of Infection, Veterinary and Ecological Sciences, The University of Liverpool, Liverpool, United Kingdom; b National Infection Service, Public Health England, Colindale, London, United Kingdom; c Division of Infection and Immunity, The Roslin Institute and Royal (Dick) School of Veterinary Studies, University of Edinburgh, Edinburgh, United Kingdom; University of Maryland School of Medicine

**Keywords:** AMR, MSM, PacBio, persistent infection, *Shigella*, UK, WGSA, genomics

## Abstract

Shigellosis is a diarrheal disease caused mainly by Shigella flexneri and Shigella sonnei. Infection is thought to be largely self-limiting, with short- to medium-term and serotype-specific immunity provided following clearance. However, cases of men who have sex with men (MSM)-associated shigellosis have been reported where *Shigella* of the same serotype were serially sampled from individuals between 1 and 1,862 days apart, possibly due to persistent carriage or reinfection with the same serotype. Here, we investigate the accessory genome dynamics of MSM-associated S. flexneri and S. sonnei isolates serially sampled from individual patients at various days apart to shed light on the adaptation of these important pathogens during infection. We find that pairs likely associated with persistent infection/carriage and with a smaller single nucleotide polymorphism (SNP) distance, demonstrated significantly less variation in accessory genome content than pairs likely associated with reinfection, and with a greater SNP distance. We observed antimicrobial resistance acquisition during *Shigella* carriage, including the gain of an extended-spectrum beta-lactamase gene during carriage. Finally, we explored large chromosomal structural variations and rearrangements in seven (five chronic and two reinfection associated) pairs of S. flexneri 3a isolates from an MSM-associated epidemic sublineage, which revealed variations at several common regions across isolate pairs, mediated by insertion sequence elements and comprising a distinct predicted functional profile. This study provides insight on the variation of accessory genome dynamics and large structural genomic changes in *Shigella* during persistent infection/carriage. In addition, we have also created a complete reference genome and biobanked isolate of the globally important pathogen, S. flexneri 3a.

## Abstract (precis)

*Shigella* spp. are Gram-negative bacteria that are the etiological agent of shigellosis, the second most common cause of diarrheal illness among children under the age of five in low-income countries. In high-income countries, shigellosis is also a sexually transmissible disease among men who have sex with men.

## INTRODUCTION

Shigellosis is a fecal-orally transmitted disease that causes dysentery and is responsible for over 164,000 deaths a year (1). In low-income countries, shigellosis is among the leading cause of moderate to severe diarrhea in children under the age of 5 years (2), whereas in high-income countries, cases are often linked to foreign travel and sexual transmission among men who have sex with men (MSM) (3–5). The causative agent of shigellosis is *Shigella*, a genus of Gram-negative bacteria comprising of four species. Among these, Shigella flexneri and S. sonnei contribute to the greatest disease burden globally. The phylogeny of S. flexneri encompasses seven genetically distinct subtypes referred to as phylogroups (PGs) (6), and S. sonnei is comprised of five subtypes referred to as lineages (7, 8). Traditionally, *Shigella* is also subdivided into serotypes that are defined by the lipopolysaccharide O-antigen structure, with S. flexneri comprising 15 serotypes and subserotypes and S. sonnei comprising a single serotype (9). Historically, S. flexneri serotype 2a has been the most prevalent serotype across the globe (10).

Endemic shigellosis among United Kingdom (UK) MSM is caused by diverse *Shigella* subtypes, giving rise to a substantial disease burden, (albeit only a fraction of the global shigellosis burden). Between 2004 and 2015, there were 3,105 domestically acquired shigellosis cases in males reported in England; of these, 77% were due to either S. sonnei, S. flexneri 2a, or S. flexneri 3a (4). Phylogenetic analyses have revealed further diversity within these serotypes, with two sublineages of S. flexneri 2a (belonging to PG3), a monophyletic lineage of S. flexneri 3a (belonging to PG2), and five clades of S. sonnei (belonging to lineage III) having cocirculated among MSM across time and locations (3, 11, 12). Although both S. flexneri serotypes cause disease and belong to distinct phylogroups, only one complete reference genome for S. flexneri 3a currently exists.

Genomic epidemiological analyses have also highlighted the importance of antimicrobial resistance (AMR) in driving MSM-associated shigellosis. For instance, although the current recommended treatment for shigellosis is ciprofloxacin, *Shigella* spp. with mutations in the quinolone resistance-determining region (QRDR) conferring resistance or reduced susceptibility to fluoroquinolones, are widely reported in MSM-associated outbreaks globally (5, 13, 14). Also, they have been responsible for driving persistent transmission of MSM-associated S. sonnei shigellosis in the UK (12). In addition to this vertically inherited AMR, the approximate doubling of MSM-associated S. flexneri 2a and S. sonnei infections between 2012 and 2014 (4) was associated with the horizontal acquisition of pKSR100, an azithromycin resistance plasmid, which enhanced the epidemics (11). This evidence of horizontal gene transfer (HGT) among shigellae and cocirculation of diverse subtypes suggests that AMR is transferred, potentially through intermediary bacterial hosts or human host coinfection with different *Shigella* subtypes, which has been reported in the Netherlands (15).

The probability of coinfection with different *Shigella* types would be increased if chronic infections occurred, for which there is emerging evidence (16). In addition, coinfection with HIV is common among shigellosis-affected MSM (with 75% being HIV infected) (13), and this could be a risk factor for sustaining prolonged infection among MSM (17, 18). Although *Shigella* infection is typically self-limiting, with infection times ranging between 1 and 4 weeks and followed by immunity against the homologous serotype (19) for a period of approximately 5 months to 2 years (20–22), this may not be the case in MSM. Serial isolation of the same *Shigella* serotype from an individual for up to 1,862 days has been reported among UK MSM (16). Due to the rarity of serial samples, the pathogen factors that may contribute to adaptation of *Shigella* during infection remain poorly characterized. This knowledge gap is shared with many other *Enterobacteriaceae* bacteria despite the importance of the family as pathogens. Thus, although the absolute number of cases of shigellosis among MSM are a fraction of the global burden, such cases present a valuable opportunity to study the adaptation of *Shigella* over the course of infection.

The intensification of MSM-associated shigellosis in England over recent years has provided a unique and diverse data set of *Shigella* isolates serially sampled from 165 individual male patients (16). Although previous analyses of SNP distances among a subset of these serially isolated *Shigella* pairs has provided valuable distance metrics for differentiating between carriage and reinfection (16), characterizing large-scale genomic changes during in-host evolution also provides important insights for understanding adaptation for prolonged infection, as has been described for Pseudomonas aeruginosa in chronic infections (23, 24). Thus, we here extend the comparisons among serially isolated *Shigella* pairs to perform detailed comparative genomic analyses and investigate the changes in shigellae over the course of long-term infection. We characterize accessory genome dynamics, including the gain and loss of AMR determinants, and compare and contrast these changes between pairs that represent long-term carriage to those that arose from reinfection. We further the study by long-read sequencing additional pairs of S. flexneri 3a to compare large-scale structural variation across the chromosome and detect signatures of conservation in genes with various metabolic functions. In performing this study, we have also generated a high-quality reference genome and publicly accessioned an isolate of the globally important pathogen S. flexneri 3a.

## RESULTS

### Change in accessory genome over time among carriage- and reinfection-associated pairs.

In order to extend our understanding of accessory genome dynamics during the course of *Shigella* infection, we examined the difference in accessory gene content between 38 pairs of carriage-associated (14 S. flexneri 2a, 9 S. flexneri 3a, and 15 S. sonnei) and 19 pairs of reinfection-associated (5 S. flexneri 2a, 6 S. flexneri 3a, and 8 S. sonnei) isolates (see Table S1 in the supplemental material). Here, we assigned the pair class (i.e., carriage or reinfection) based on pairwise SNP distances and sampling time interval of the pairs, as described in Materials and Methods. First, we assessed the correlation between pairwise SNP distances and pairwise gene content variation, wherein pairwise gene content is defined as the total number of unique genes within a pair that were present in one isolate but not in the other, incorporating both gain and loss over time. This revealed a positive correlation between the two variables that is statistically significant (*P* < 0.05) for all three *Shigella* serotypes, although the association is stronger for S. sonnei (*r *=* *0.80, Spearman’s rank correlation coefficient) than for S. flexneri 2a (*r *=* *0.56) and S. flexneri 3a (*r *=* *0.65) (see Fig. S1). These data suggest that as the core genome distance (chromosomal SNPs) between a pair of serially sampled isolates increases, the accessory genome distance also increases.

10.1128/mBio.00254-21.1 FIG S1 Association of gene content variation with SNP distance for 19 S. flexneri 2a (A), 15 S. flexneri 3a (B), and 23 S. sonnei (C) pairs of isolates sampled at two time points. Each point in the scatter plot represents a pair of isolates. Pairs are colored according to classification, carriage association (red) and reinfection association (green). The pairwise gene content variation is plotted along the *y* axis and is the total number of unique genes between a pair of isolates. The pairwise SNP distance between each pair is plotted along the *x* axis. Spearman’s rank coefficients were used to assess the correlation between the two variables for each serotype and are displayed with the *P* value on the right-hand side of each plot. Download FIG S1, TIF file, 1.5 MB. © Crown copyright 2021. 2021 Crown https://creativecommons.org/licenses/by/4.0/ This content is distributed under the terms of the Creative Commons Attribution 4.0 International license.

10.1128/mBio.00254-21.5 TABLE S1 Number of isolate pairs analyzed in the present study, broken down by *Shigella* subtypes and classification as carriage associated or reinfection associated. Download Table S1, DOCX file, 0.01 MB. © Crown copyright 2021. 2021 Crown https://creativecommons.org/licenses/by/4.0/ This content is distributed under the terms of the Creative Commons Attribution 4.0 International license.

**FIG 1:**
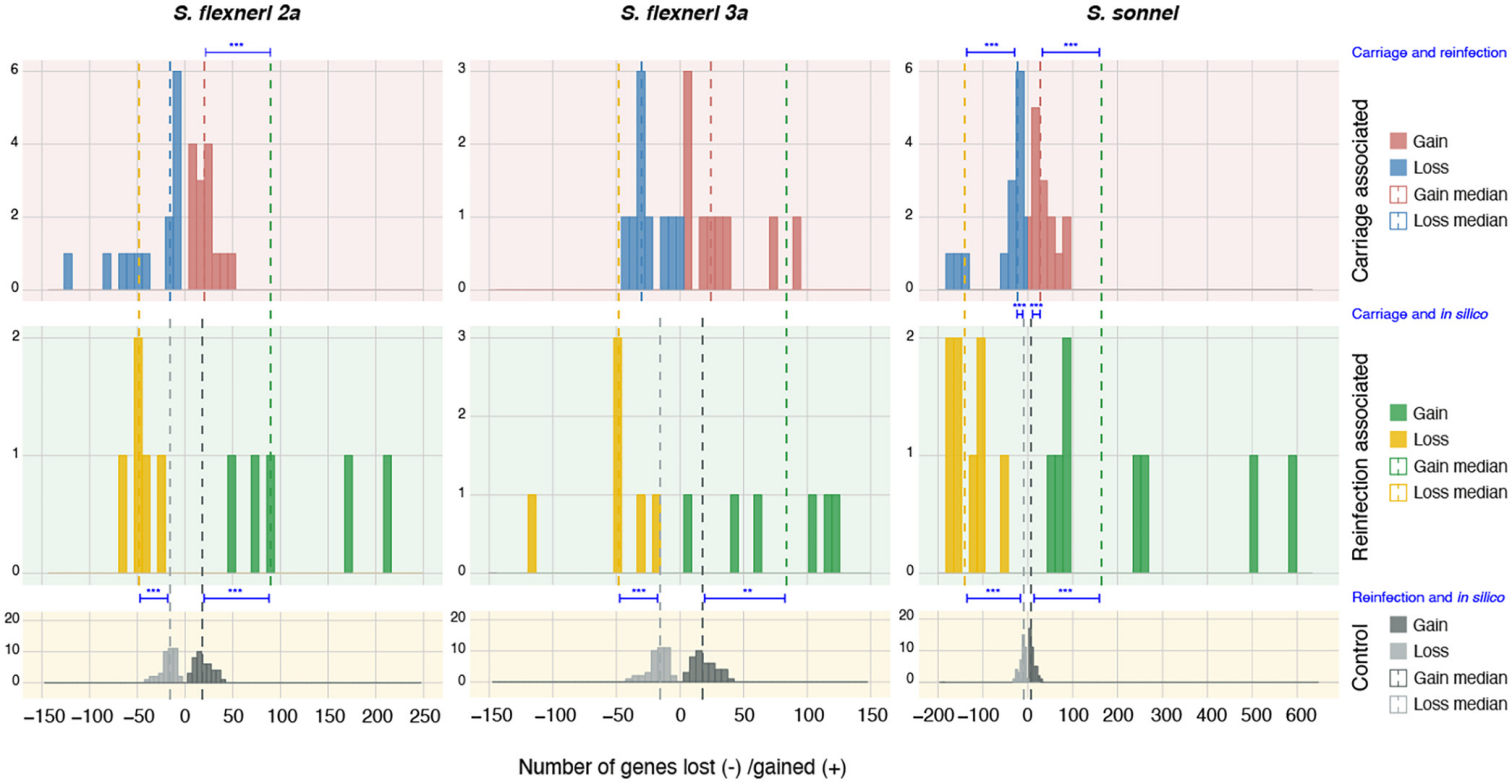
Gene content variation comparison of *Shigella* isolate pairs by pair class and *Shigella* subtype. Frequency histogram plots show the frequency of isolate pairs (*y* axis) with various levels of genes lost and gained (*x* axis, with negative values showing genes lost and positive values showing genes gained). Different *Shigella* subtypes are displayed separately (S. flexneri 2a [left], S. flexneri 3a [middle], and S. sonnei [right]), as are different pair classes (carriage-associated pairs along the top row, reinfection-associated pairs along the middle row, and *in silico* control pairs showing intragenome stochastic variation due to *in silico* processes along the bottom row). The rightmost color keys show the distributions (in histograms) and medians (dashed vertical lines) of genes gained or lost for each *Shigella* subtype and pair class that formed the basis for statistical comparisons. Statistical comparisons between these distributions (carriage versus reinfection, *in silico* versus carriage, and *in silico* versus reinfection) were performed using Mann-Whitney U tests, where the *P* value was <0.05. Comparator bars between median values are shown interleaved in histograms with asterisks representing the *P* value (*, *P* < 0.05; **, *P* < 0.005; ***, *P* < 0.0005). Labels of the according statistical comparisons are displayed on the right in blue.

Next, we examined the effect of pair class to disentangle the variations contributed by gain and loss events. Here, we define “gain” as genes present in the second and absent in the first isolate in a pair; “loss” denotes genes that were present in the first but absent in the second isolate. This analysis revealed that reinfection-associated pairs generally had a greater distribution in the number of genes gained and lost compared to carriage-associated pairs (Fig. 1). The greatest distribution was observed in S. sonnei reinfection-associated pairs, with the number of genes lost ranging from 57 to 182 and genes gained ranging from 47 to 597. The numbers of genes gained and lost were consistently higher for reinfection-associated pairs compared to carriage-associated pairs for all serotypes (Fig. 1). However, a *P* value of <0.05 between carriage and reinfection pairs was only observed for the number of genes gained in S. flexneri 2a (*P* = 0.0016, Mann-Whitney U test), and both genes gained (*P* = 0.0009) and lost (*P* = 0.0033) in S. sonnei. Thus, the accessory genome diversity accumulated over the course of infection in carriage pairs was lower than that between different *Shigella* infecting a single individual on reinfection.

We also performed an *in silico* control to assess whether the observed variations in gene contents among isolate pairs were biological, rather than the result of stochastic variation (e.g., in genome assembly, annotation, and clustering). Specifically, we assembled genomes from synthetic read sets of varied length and insert size generated from a reference genome for each species (20BP for S. flexneri and Ss046 for S. sonnei), which revealed various numbers of coding sequences (CDS), ranging from 4,215 to 4,234 for 20BP and from 4,228 to 4,247 for Ss046 (see Table S2). We also repeated pairwise homologous sequence comparison with the synthetic draft genomes, as described above, and observed considerable gene content variation (Fig. 1). Distributions of the gene content variation generated from the *in silico* control was statistically compared to the distributions of carriage- and reinfection-associated pairs. Significant increases (indicating true biological variation) in gene gain and loss were observed for all reinfection-associated pairs (*P* < 0.005), as well as for S. sonnei carriage-associated pairs (*P* < 0.0008) (Fig. 1). This provides supporting evidence of significant accessory genome change in S. sonnei carriage pairs but inadequate support for S. flexneri carriage pairs.

10.1128/mBio.00254-21.6 TABLE S2 Number of CDS annotated from draft genome assemblies generated from synthetic reads of various length and insert size of 20BP and Ss046 complete reference genomes. Download Table S2, DOCX file, 0.01 MB. © Crown copyright 2021. 2021 Crown https://creativecommons.org/licenses/by/4.0/ This content is distributed under the terms of the Creative Commons Attribution 4.0 International license.

### Gain or loss of AMR genes and known mobile genetic elements.

Since AMR is increasing among *Shigella* spp., we screened for the prevalence of genetic determinants that confer resistance, including horizontally acquired genes and point mutations. As expected, all S. flexneri and S. sonnei isolates within the dataset were multidrug resistant, harboring genetic determinants conferring resistance to three or more antimicrobial classes (see Fig. S2 in the supplemental material). The most commonly detected genes in S. flexneri (present in >90% of the isolates) were *aadA1*, *blaEC*, *catA1*, and *tetB* encoding aminoglycoside, beta-lactam, phenicol, and tetracycline resistance, respectively. For S. sonnei, the most commonly detected genes (present in 100% of isolates) were *blaEC*, *sat2*, and *dfrA1* encoding beta-lactam, streptothricin, and trimethoprim resistance, respectively.

10.1128/mBio.00254-21.2 FIG S2 Frequency of AMR genes and AMR genotypic profiles among S. flexneri (A) and S. sonnei (B) serially isolated from MSM. Each vertically connected black dot in the combination matrix in the center panel represents a unique genotypic AMR profile, in which a black dot represents the presence of a resistance determinant. The leftmost horizontal bar plot shows the number of AMR genes and QRDR mutations detected, grouped according to drug classes. The uppermost vertical bar plot shows the number of isolates exhibiting the genotypic AMR profile, with the exact number of isolates displayed above the bars. Download FIG S2, TIF file, 2.3 MB. © Crown copyright 2021. 2021 Crown https://creativecommons.org/licenses/by/4.0/ This content is distributed under the terms of the Creative Commons Attribution 4.0 International license.

We assessed for presence of AMR mobile genetic elements (MGEs) commonly found in MSM-associated *Shigella*, including the pKSR100 plasmid. This plasmid carries the AMR genes *mphA* and *ermB*, which confer high-level resistance to azithromycin, and is associated with driving the success of *Shigella* epidemics in MSM (3, 5, 11, 25). Short-read mapping confirmed this resistance plasmid was present in 82% (31/38) of S. flexneri 2a, 70% (21/30) of S. flexneri 3a, and 43% (20/46) of S. sonnei isolates. Other horizontally transmissible elements, including the chromosomal *Shigella*-resistance locus multidrug resistance element (SRL-MDRE), carrying the resistance genes *tetB*, *bla*_OXA-1_, and *catA1* were identified in 94% (64/68) of S. flexneri isolates (36 S. flexneri 2a and 28 S. flexneri 3a) (see Table S3). For S. sonnei, all isolates carried the chromosomal transposon Tn*7* and class II integron (In2) that contain the resistance genes *aadA1* and *dfrA1*. Furthermore, the presence of the small plasmid spA, which carries *sul2*, *strAB*, and *tetA*, was identified in 65% (30/46) of S. sonnei isolates. In addition, pCERC1, which carries *dfrA14*, *sul2*, and *strAB*, was detected in 7% (4/68) of S. flexneri (one S. flexneri 2a and three S. flexneri 3a) isolates (see Table S3). In addition to investigating known MGEs, we explored presence of other associated MGEs (specifically plasmid replicons) on AMR gene-containing contigs. This approach identified no additional replicons, so the high prevalence of AMR genes and genotypic resistance profiles among the isolate pairs is consistent with previous descriptions of UK MSM-associated *Shigella* sublineages and is largely defined by known mobile genetic elements.

10.1128/mBio.00254-21.7 TABLE S3 Information regarding time interval between serial isolation, pair classification, and AMR genetic determinants detected among S. flexneri and S. sonnei isolate pairs. AMR genetic determinants are grouped according to the known plasmids associated with the carriage of the determinants. Download Table S3, XLSX file, 0.02 MB. © Crown copyright 2021. 2021 Crown https://creativecommons.org/licenses/by/4.0/ This content is distributed under the terms of the Creative Commons Attribution 4.0 International license.

To investigate changes in AMR over time, we explored which resistance genes were gained and lost over the course of infection. Here, we applied the working definition of gained and lost used above. Discrepancies in AMR genes between a pair of isolates were observed among 18 of 57 pairs, 10 S. flexneri (7 S. flexneri 3a and 3 S. flexneri 2a) and 8 S. sonnei isolates, reflecting population level trends (Table 1). Specifically, these variations were mainly contributed by the gain or loss of known AMR-associated MGEs. The acquisition of the pKSR100 plasmid was often observed in reinfection-associated pairs, whereas the pCERC1 plasmid was often lost in both carriage- and reinfection-associated pairs. These individual trends of pKSR100 gain and pCERC1 loss are consistent with observations across MSM-associated shigellae (3, 11). The spA plasmid was also gained in three S. sonnei reinfection pairs. Concerningly, there was evidence of AMR gain in two carriage-associated pairs, with the extended spectrum beta-lactamase gene *bla*_SHV-12_ being acquired by an S. flexneri 2a (Case ID I) pair and the broad-spectrum beta-lactamase gene *bla*_TEM-1_ being gained in an S. sonnei pair (case ID L) (Table 1), suggesting AMR acquisition during carriage. A BLASTn search of the 52,219-bp contiguous sequence carrying the *bla*_TEM-1_ gene revealed 86% coverage and 99% identity with an Escherichia coli O182:H21 plasmid (GenBank accession number CP024250.1), while the length of the contig carrying *bla*_SHV-12_ spanned only the length of the gene, precluding further speculation on the origin of this gene.

**TABLE 1:** Variation in AMR genes detected among paired S. flexneri 2a, S. flexneri 3a, and S. sonnei isolates

Reinfection or carriage	Species	Case ID^*b*^	Interval (days)	MDR plasmid(s)^*a*^		Associated AMR genetic determinant(s)
				Gained	Lost	
Carriage	S. flexneri 3a	F	27		pCERC1	*dfrA14*, *sul2*, *strAB*
		O	83		pCERC1	*sul2*, *strAB*
	S. flexneri 2a	I	6			*bla*_SHV-12_
	S. sonnei	L	35			*bla*_TEM-1_
Reinfection	S. flexneri 2a	A	1,142	pKSR100		*mph*(*A*), *bla*_TEM-1_, *dfrA17*, *sul1*, *aadA5*
		C	496		pCERC1	*dfrA14*, *sul2*, *strAB*
	S. flexneri 3a	C	805	pKSR100		*erm*(*B*), *mph*(*A*), *bla*_TEM-1_
		E	193		pKSR100 integron	*dfrA17*, *sul1*, *aadA5*
		I	1,862	pKSR100		*erm*(*B*), *mph*(*A*), *bla*_TEM-1_
		D	1,099		pCERC1	*dfrA14*, *sul2*, *strAB*
		J	905	pKSR100	pCERC1	*mph*(*A*), *bla*_TEM-1_, *dfrA17*, *sul1*, *aadA5*, *dfrA14*, *sul2*, *strAB*
	S. sonnei	B	925	spA		*strAB*, *sul2*, *tetA*
		C	1409	spA, pKSR100		*strAB*, *sul2*, *tetA*, *bla*_TEM-1_, *erm*(*B*), *aadA5*, *dfrA17*, *sul1*, *mph*(*A*)
		D	42		spA	*strAB*, *sul2*, *tetA*
		G	1,208	spA, pKSR100		*strAB*, *sul2*, *tetA*, *bla*_TEM-1_, *erm*(*B*), *aadA5*, *dfrA17*, *sul1*, *mph*(*A*)
		I	659	pKSR100		*bla*_TEM-1_, *erm*(*B*), *mph*(*A*)
		J	481	pKSR100		*bla*_TEM-1_, *erm*(*B*), *mph*(*A*)
		V	184			*aadA1*

a “Plasmid gained” is defined as the plasmid being present in the later but absent in the earlier-sampled isolate of a pair; “plasmid lost” is defined as the plasmid being absent in the later isolate but present in the earlier-sampled isolate of a pair. MDR, multidrug resistant.

b Further metadata on isolate pairs are provided in Table S3.

Point mutations in the QRDR were identified in 65% (30/46) of S. sonnei isolates, 19 of which had triple mutations (*gyrA* S83L, *gyrA* S87G, and *parC* S80I) known to confer resistance against ciprofloxacin, and 10 had a single mutation (*gyrA* S83L or D87G) conferring reduced susceptibility (see Table S3). QRDR point mutations were rare in S. flexneri, with only a single *gyrA* S83L mutation detected in only two S. flexneri 3a isolates. Although the rates of quinolone resistance were moderate in S. sonnei and low in S. flexneri, there was no sign of *de novo* mutation in the QRDR region over the course of infection since we did not observe any carriage-associated pairs that acquired QRDR mutations.

### Generation of an *S. flexneri* 3a isolate reference genome and classification of carriage- or reinfection-associated pairs.

**FIG 2:**
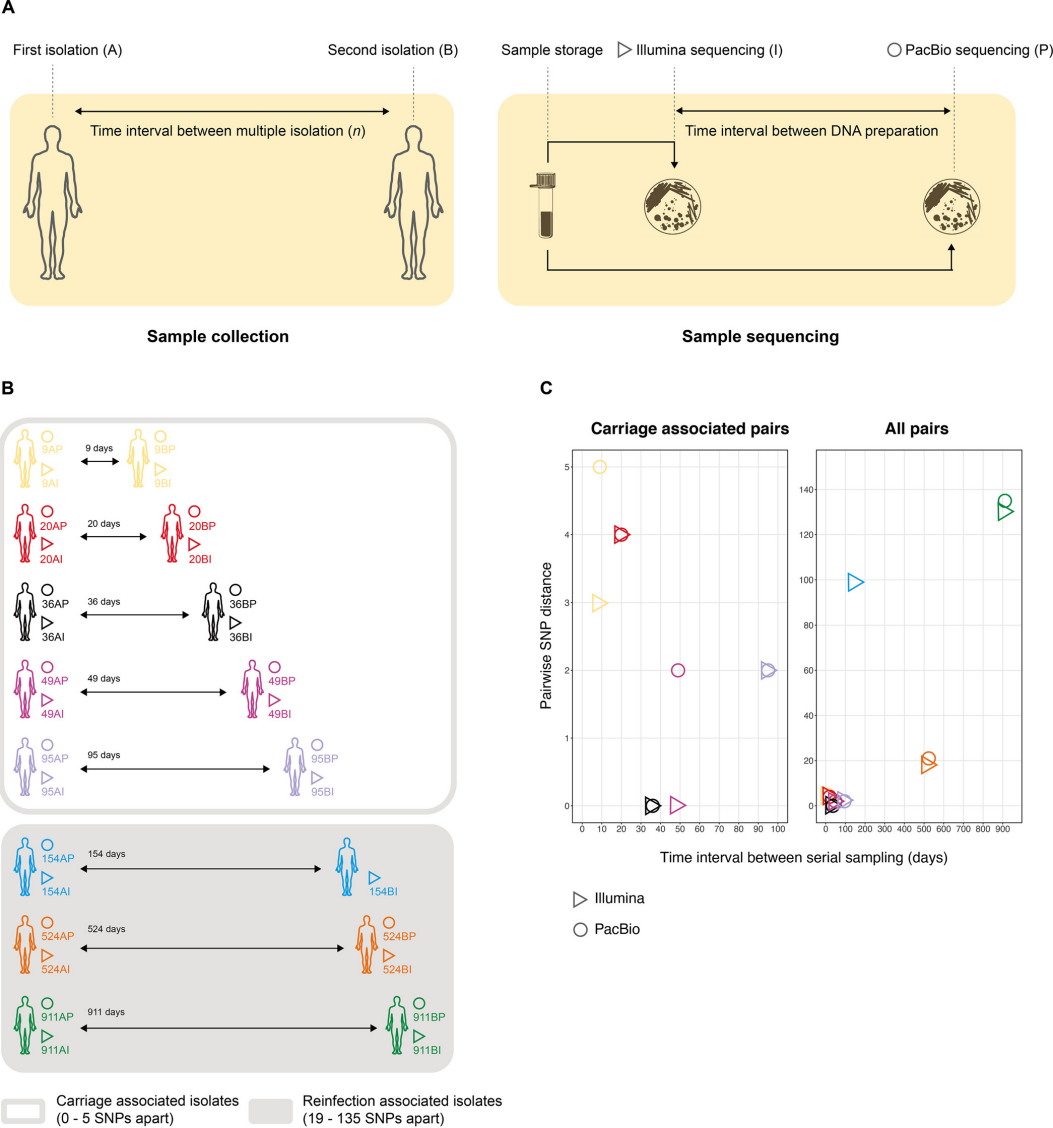
Isolate pair processing, genome notation, and SNP distance by time interval, as estimated by different technologies in S. flexneri 3a structural variation substudy. (A) Isolates from each pair were serially sampled from the same patient (time point notation A/B). Isolates were stored and later Illumina sequenced and, after a considerable time interval (e.g., several years), revived and DNA prepared for PacBio sequencing (technology notation I/P). (B) Serial isolation of S. flexneri 3a was performed from eight patients sampled between 9 and 911 days apart (numerical time interval notation). The names of the 31 genome sequences used in the present study are presented in the diagram according to this combined notation. (C) The scatterplots display pairwise SNP distances between serial isolate pairs, as estimated by either Illumina (triangles) or PacBio (circles) sequencing. A given colored symbol represents the pairwise SNP distance between genome sequences (from B) that share a same-color symbol. For example, the yellow triangle represents the pairwise distance between genome sequences 9AI and 9BI, while the green circle represents the pairwise distance between 911AP and 911BP. The scatterplot on the left displays pairwise distances among carriage-associated pairs, and the plot on the right across all isolate pairs analyzed in this study.

To determine the structural variation and genome rearrangement of S. flexneri over time, we PacBio sequenced 16 isolates from an epidemic sublineage of MSM-associated S. flexneri 3a serially isolated from eight individuals at two time points with time intervals ranging 9 to 911 days apart (Fig. 2A and B). PacBio sequencing of one isolate (154BP) failed, so only seven isolate pairs were further examined. A complete genome for isolate 20BP was generated, which comprised a 4,522,047-bp chromosome, a 231,165-bp virulence plasmid, and a 72,593-bp pKSR1000 plasmid. This complete genome was then used as a high-quality reference genome for further analyses and has been deposited in NCBI under accession number GCA_904066025. The cognate isolate is NCTC 14607.

We assessed pairwise SNP distances of the seven S. flexneri 3a pairs by mapping all isolate genomes to the 20BP reference genome and compared the SNP variants between the two isolates within each pair. We detected genetic distances ranging from 0 to 135 SNPs apart and, generally, pairwise SNP distances increased with time interval between serial isolations (Fig. 2C; see also Table S4), an observation consistent with genomic epidemiological definitions published previously (16). By applying the aforementioned definitions of carriage- and reinfection-associated pairs (see Materials and Methods), we identified five carriage- and three reinfection-associated pairs.

10.1128/mBio.00254-21.8 TABLE S4 Pairwise SNP distances and time interval between serial sampling of isolates from an epidemic sublineage of MSM-associated S. flexneri 3a, sequenced using Illumina and PacBio. * The number of SNPs detected when first (A) isolate sequenced using Illumina (I) is mapped onto the draft genome of second isolate (B) sequenced using PacBio (P). ** The difference in number of SNPs called through mapping all isolates to 20BP reference genome (column D) and mapping of the first to the second isolate (column F). Download Table S4, XLSX file, 0.01 MB. © Crown copyright 2021. 2021 Crown https://creativecommons.org/licenses/by/4.0/ This content is distributed under the terms of the Creative Commons Attribution 4.0 International license.

Notably, Illumina data for the 16 S. flexneri 3a isolates were already available from a previous study (3). Since all of the isolates had been Illumina and PacBio sequenced, we used these data to examine the effect of different sequencing technologies (and/or DNA preparations) on the estimation of SNP distance between serially isolated pairs (Fig. 2). This revealed that the pairwise SNP distances estimated by the two technologies were 0 to 5 SNPs apart, which is within the commonly used epidemiological cutoff (26). For example, a pairwise SNP distance of 2 was estimated between genomes 9AI and 9BI sequenced with Illumina, while a pairwise SNP distance of 3 was estimated between genomes 9AP and 9BP sequenced with PacBio (Fig. 2C; see also Table S4). We also examined the pairwise SNP distance between each pair by mapping the genome of the second isolate against the first isolate, which revealed consistent trend of increase genetic distance in relation to time compared to reference mapping (see Table S4).

### Large-scale variation of *S. flexneri* genome over time.

**FIG 3:**
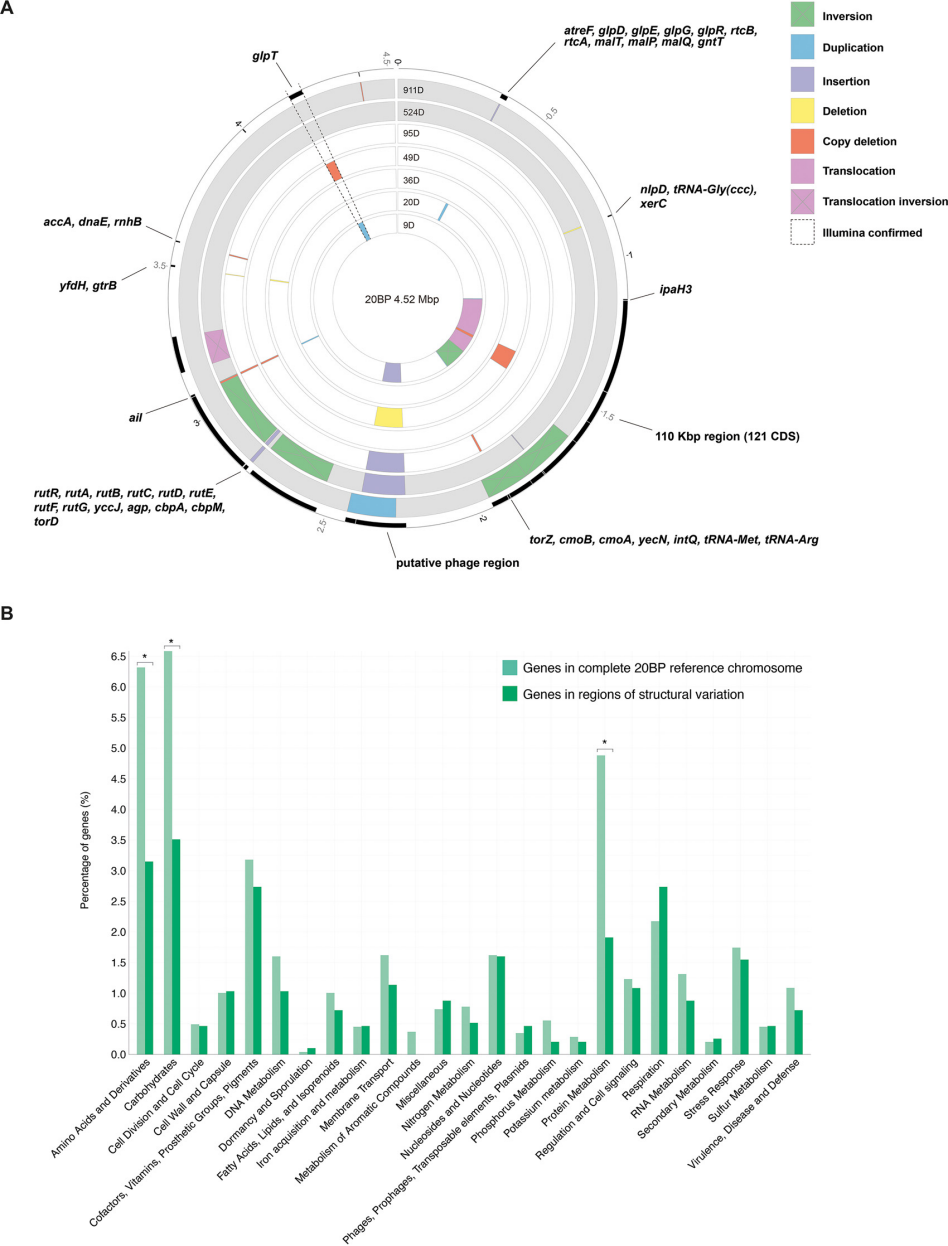
Chromosomal structural variations among MSM-associated S. flexneri 3a. (A) Genomic regions demonstrating structural variations and rearrangements among seven serial isolate pairs. All genomes were aligned to 20BP reference chromosome to detect structural variations, and the commonality or uniqueness of each variation was used to determine variation within each pair. Each concentric ring represents a pairwise genome comparison of an isolate pair sequenced with PacBio. The time interval (days) between serial sampling of each pair is displayed at the terminal edge of the rings. Genome comparisons for two reinfection pairs are colored in gray. Colored blocks are overlaid onto each comparison and indicate the nature and frame of structural variations according to the inlaid key. The outermost track (in black) displays CDS within regions of structural variations and annotated genes are labeled accordingly, genes encoding hypothetical proteins are not labeled. A genomic region with duplication and deletion events confirmed by both Illumina and PacBio sequence data are outlined by dashed lines. (B) Distribution of genes belonging to different GO categories across the reference chromosome compared with variant regions. Within each group, the light green bar represents the percentage of genes (*y* axis) predicted to belong to a particular GO category (*x* axis) identified across the entire 20BP reference chromosome. This contrasts with the dark green bar, which represents the percentage of genes belonging to the same GO category but identified exclusively within the variant regions. Genes with known function that could not be assigned to a category are shown elsewhere (see Fig. S4). Categories with significant difference (*P* < 0.05, chi-square test) between the proportion of genes between the two groups are indicated by an asterisk.

To detect structural rearrangements among the seven pairs of S. flexneri 3a, we aligned each PacBio-sequenced genomes against the 20BP reference genome and then assessed the discrepancies between each pair. Across the 7 pairs of isolates, we identified 34 structural variations across 14 genomic regions, including 9 copy deletions, 7 insertions, 7 duplications, 5 inversions, 4 deletions, 1 translocation, and 1 translocation inversion (Fig. 3A). Three structural variants were less than 1,500 bp and mapped to insertion sequence (IS) elements. We analyzed sequences at the borders of the remaining 31 variants to determine possible mechanisms facilitating the rearrangements, which revealed that 15 variations had arisen through recombination between homologous IS copies and two variants had occurred through recombination between ribosomal operons (see Table S5). Of the remaining 14 variants, 7 possessed an IS element on one end. We did not detect the presence of repeat sequences or IS elements at the borders of the remaining 7 variants, indicating that rearrangements may have been facilitated by an unknown mechanism.

10.1128/mBio.00254-21.9 TABLE S5 Location, size and type of structural variation and genome rearrangement detected among the chromosome of seven S. flexneri 3a isolate pairs sampled at various days apart. *^a^*Positions of the variants are given according to the genomic coordinates of 20BP reference genome (GCA_904066025). *^b^*RRE denotes variants with homologous IS copies identified at both ends and movements facilitated by recombination between repeat elements. SE denotes variants with an IS element identified at a single end. Identity of the IS element is indicated after the hyphen. *^c^*Genes that are identified within regions of large-scale variants are listed, for variants containing more than 10 genes only the first and last genes are listed. Download Table S5, XLSX file, 0.01 MB. © Crown copyright 2021. 2021 Crown https://creativecommons.org/licenses/by/4.0/ This content is distributed under the terms of the Creative Commons Attribution 4.0 International license.

10.1128/mBio.00254-21.4 FIG S4 Distribution of genes belonging to different GO categories, predicted by RAST. The different categories are plotted along the *x* axis, and the percentage of genes predicted to be in the category is plotted along the *y* axis. For each category, the percentage of genes predicted across the 20BP reference chromosome (light green bars) is compared to the percentage of genes predicted within the structural variable regions (dark green bars). Categories with significant difference (*P* < 0.05, chi-square test) are indicated by an asterisk. Download FIG S4, TIF file, 0.8 MB. © Crown copyright 2021. 2021 Crown https://creativecommons.org/licenses/by/4.0/ This content is distributed under the terms of the Creative Commons Attribution 4.0 International license.

Rearrangements occurring in two regions were commonly observed among the pairs, including a 166-kbp region at ∼2.24 to 2.40 Mbp and an 8-kbp region at ∼3.07 to 3.08 Mbp, identified among four pairs (three carriage and one reinfection) (Fig. 3A). The former is a region flanked by IS*91* copies at both ends and carries 207 predicted genes encoding an incomplete prophage. A reinfection-associated pair sampled 911 days apart displayed duplication that overlaps this region and offset by ∼37 kbp, also flanked by IS*91* copies. In regard to the latter, the 8-kbp region was flanked by homologous IS*1* copies and appears to fall within an intact prophage. A total of 11 genes were identified within this region, the majority (10/11) of which encode hypothetical proteins. However, a single gene is predicted as a Ail/Lom family outer membrane β-barrel protein, in which the Ail protein is a known virulence factor thought to promote host cell invasion (27) and Lom is a phage protein expressed during lysogeny (28).

To confirm duplication and deletion variations (20 of 34 total variations), we compared results from mappings of short-read Illumina data with mappings of long-read PacBio data for the same isolate (i.e., mapping of 49BI and 49BP). Consistency between short- and long-read mapping results was taken to represent a true variation between isolate pairs that likely occurred *in vivo*. In contrast, discrepancies between the two mappings might suggest that the variation was introduced by storage and/or different sequencing technology/DNA preparations. Consistency for only one region at 4.17 to 4.22 Mbp of 127 kbp was confirmed (Fig. 3A; see also Fig. S3). This region of confirmed variation was observed in two carriage-associated pairs, with a duplication and a deletion in pairs sampled 9 and 49 days apart, respectively (Fig. 3A). This region is flanked by rRNA operons and contains 37 CDS, including *ompA*, which encodes the outer membrane protein A, a virulence factor involved in facilitating cell-to-cell spread and a target for vaccine development (29, 30).

10.1128/mBio.00254-21.3 FIG S3 Short and long read mapping of a 127-kbp region at 4.17 to 4.22 Mbp. (A) Reads of isolates sampled at 9-day intervals were mapped against complete reference sequence of 20BP, which confirmed duplication of the region in the isolate sampled at the second time interval. (B) Reads of isolates sampled at 49-day intervals confirmed copy deletion of the same region in the isolate sampled at the second time interval. Download FIG S3, TIF file, 2.4 MB. © Crown copyright 2021. 2021 Crown https://creativecommons.org/licenses/by/4.0/ This content is distributed under the terms of the Creative Commons Attribution 4.0 International license.

A total of 1,791 genes were located within genomic regions demonstrating structural variations and rearrangements. In order to determine whether particular gene functions were enriched within these genomically plastic regions, we annotated the genes found exclusively within these regions and assigned them to predicted functional categories according to gene ontology (GO) categories. The same was done for all genes identified across the 20BP reference chromosome. Then, a chi-square test was used to test for statistical difference for the percentage of genes within each GO category, between the variable regions, and across the entire reference chromosome. This revealed majority of genes (59%) across the 20BP reference chromosome were predicted with unknown function and were not assigned a GO category (see Fig. S4). Interestingly, there was a significant increase (*P* < 0.00001) in the proportion of genes with unknown function in structural variable regions, which accounted for 72% of all genes predicted in these regions. Aside from this, significant differences in the percentage of genes belonging to three functional categories were observed, all being depleted across the structurally variable regions (Fig. 3B). Specifically, these regions contained a lower proportion of genes predicted to function in the synthesis and metabolism of amino acids and derivatives, in the metabolism of carbohydrates, and in the metabolism of proteins.

## DISCUSSION

Infection with *Shigella* spp. is traditionally thought to be self-limiting with protective immunity acquired against homologous serotype following clearance of the infection (19). However, this view has been challenged by recent observations of patients being diagnosed with secondary isolation of the same serotype, within the expected time frame of immunity (16). To further our understanding of the large-scale genomic changes occurring in these infections, we extended previous study on SNP and phylogenetic analysis of serially isolated pairs of MSM-associated S. flexneri 2a, S. flexneri 3a, and S. sonnei (16). It is worth noting that, although we have used working definitions of carriage and reinfection for narrative, as defined by SNP distance and time intervals between pairs, serial isolations from individuals may represent multiple disease phenomena among MSM. Other factors, such as coinfection with HIV and high contact rates within the transmission network, could also have an effect on altering individual immune function and cause prolonged infection times, relapsing, or reinfection of immunocompromised or healthy individuals with the same serotype (3, 31). Thus, further prospective clinical and epidemiological work is required to fully differentiate between persistent carriage or chronic infection, as well as reinfection between closely or more distantly related isolates, which is under way.

Nonetheless, inspection of changes in the accessory genome among the isolate pairs revealed several interesting observations. First, there was an association between SNP distance and the magnitude of unique gene content variation, where reinfection-associated pairs had greater SNP distances and varied by a greater number of genes than did carriage-associated pairs, although the association was weak for S. flexneri. This observation was in agreement with previous work showing that secondary isolations from an individual are probably caused by a different serotype or a more distantly related isolate (16). Second, the average number of genes gained and lost between the pair class (i.e., carriage and reinfection) differed, but this was not uniform across the three serotypes. In the case of S. sonnei, reinfection-associated pairs demonstrated on average a greater number of genes gained and lost than carriage-associated pairs. This further supports the finding of a decreased genetic distance among carriage pairs compared to reinfection-associated pairs. It is important to note that the lack of statistical significance detected in S. flexneri pairs may have been due to a smaller sample size of S. flexneri 2a (*n *=* *19) and S. flexneri 3a (*n *=* *15) compared to S. sonnei (*n *=* *23). Finally, there were considerable differences in the number of accessory genes gained and lost in S. sonnei carriage-associated pairs, suggesting that a significant degree of within-host evolution might be occurring. Previous studies of chronic bacterial infections have suggested that a combination of microevolution and large genetic changes contributes to pathogen adaptation, an important factor associated with prolonged infection (32). For example genomic changes in Pseudomonas aeruginosa during chronic infection of patients with cystic fibrosis have shown that while *de novo* mutation is considered an import evolutionary adaptative process (33), the deletion and the acquisition of genes is also an important factor (23, 24). Thus, in order to gain a further insight into the adaptation of bacteria over the course of infection, it is important to also consider the accessory genome dynamics. This is especially true for *Shigella* and other bacterial species with highly plastic genomes, which exist in complex environments with diverse microbial communities and challenges from the host immune system.

MSM-associated shigellosis is intimately associated with increasing AMR (3), and gain of resistance through HGT is known to enhance and drive MSM-associated *Shigella* epidemics (11, 12, 25). Here, we observed the acquisition of two different beta-lactamase genes (one extended spectrum) in two carriage-associated pairs, one S. flexneri 2a and one S. sonnei. Since we were unable to fully reconstruct the genetic context for these genes, we could not identify their potential origin, although a contig carrying the *bla*_TEM-1_ gene in S. sonnei shared a high level of similarity with an E. coli plasmid, and HGT of AMR plasmids between *Shigella* and E. coli in the gut has been previously suggested (34–36). Thus, the acquisition of this resistance gene could have been facilitated by HGT from E. coli or other bacterial species in the gut. Since evolution of drug resistance within a host is a well-described adaptive feature of bacteria such as P. aeruginosa (37) and Mycobacterium tuberculosis during chronic infection (38), the acquisition of genetic determinants of AMR in two carriage-associated pairs likely indicates a similar adaptation in shigellae during infection. Furthermore, while the acquisition of AMR in *Enterobacteriaceae* is well documented in hospital and care facilities (39–41), we have demonstrated this here in a community setting, among a patient subpopulation frequently undergoing antimicrobial treatment (13, 42, 43). This mechanism of resistance acquisition in *Shigella* during chronic infection may be similar to those acting in other multidrug-resistant *Enterobacteriaceae*, such as uropathogenic Escherichia coli and Klebsiella pneumoniae, which can persist for a long period of time, and patients may undergo repeat antibiotic treatments due to chronic and recurrent infections (44).

In addition to accessory genes, we extended the study to look at large chromosomal structural variations and rearrangements during *Shigella* carriage, which are known to play an important role in shaping the evolution of the pathogen (45). We PacBio sequenced seven S. flexneri 3a pairs (five carriage and two reinfection) from an MSM-associated epidemic sublineage and complemented the analyses with available Illumina data. This revealed few regions exhibiting genuine structural variations over the course of patient infection. Interestingly, the majority of the structural variations observed appeared to be artefactual, resulting from either prolonged storage or different DNA preparations/sequencing technologies. Remarkably, however, some regions of artefactual variation were common among isolate pairs, and our rich genome data set allowed us to disentangle some factors potentially contributing to the artifact. For example, a prominent large region of variation common across pairs was a 166-kbp prophage region in five PacBio-sequenced genomes. The absence of this region exclusively in the PacBio-sequenced genomes may have resulted from their prolonged storage (relative to the Illumina preparations) through discarding genes with dispensable functions in the storage environment (46, 47). Despite this being artefactual, the observation that this prophage region is lost in storage and retained in the clinical setting suggests that the region may have important functions involving infection and/or ecological interaction and thus warrants further investigation.

Isolates used in this study were stored in darkness, at ambient temperature, on Dorset’s egg medium at the Gastrointestinal Bacterial Reference Unit (GBRU) reference laboratory. Half of the variants detected here were flanked by IS elements, which previously have been demonstrated to contribute to large genome rearrangements of resting E. coli K-12 stored in agar stab cultures kept at room temperature (48). Furthermore, long-term storage of E. coli kept in the same condition revealed considerable genetic instability, suggesting that the genomes of resting bacteria are more dynamic than previously believed (49). Thus, the lack of confirmation in the majority of variants detected here highlight the importance of considering the effect of storage and the need for due caution when examining large-scale genomic rearrangements of archived bacteria stored under such conditions. Finally, we observed a significantly lower proportion of genes involved in key metabolic processes, including amino acid (and derivatives), carbohydrate, and protein metabolism, present in the variable regions relative to the entire chromosome. Since large structural rearrangements can be particularly deleterious and are often purged by purifying selection, constraining genome rearrangement at certain regions is an evolutionary strategy to help conserve essential genes in stable regions across the chromosome (50). Thus, the selective depletion of genes with these key metabolic processes in variable regions suggests that these may be functionally important for *Shigella* survival. Alongside the depletion of known metabolic genes, there was a comparative enrichment of genes of unknown function in variable regions, highlighting yet again the limitations of our current understanding of the functional pathways of this important pathogen.

In summary, we utilized isolate pairs occurring in a comparatively new infection setting for *Shigella* to characterize accessory genome dynamics during persistent infection. We showed an overall gain of AMR across isolate pairs, including during carriage, and consistent with population trends. We also detected genuine and artefactual variations, both of which may have biological relevance, and this should also act as a warning for future studies of structural variation of archived bacteria. Notably, due to the limited sampling intervals and methodology applied, we have not captured all possible variations (i.e., transient, small, and single gene variants). However, we have provided novel insights to large-scale genomic variations in *Shigella* over time, an important step in understanding how this pathogen adapts during infection and of potential broader relevance for the important pathogen family *Enterobacteriaceae*. To this end, we have also deposited the cognate strain for the 20BP reference genome of the intercontinentally transmitting S. flexneri 3a in the National Collection for Type Cultures (NCTC).

## MATERIALS AND METHODS

### Isolates with routinely generated Illumina sequencing data.

Short-read genome sequencing data of *Shigella* used to characterize accessory genome dynamics were generated as part of routine national surveillance by Public Health England (51, 52) and retrieved from the National Center for Biotechnology Information (NCBI) under the BioProject PRJNA315192. A total of 114 isolates were included in the present study; these were classified as arising from carriage or reinfection of individuals based on genomic epidemiological analysis of core SNP phylogenies in Allen et al. (16). This data set comprised a subset (57/85) of S. flexneri 2a, S. flexneri 3a, and S. sonnei isolate pairs collected between 2012 and 2018 from adult males diagnosed with multiple isolates of the same serotype for which whole-genome sequencing data were available (see Table S6). All 34 of the S. flexneri and more than half of the S. sonnei (14/23) pairs from this data set belonged to previously described epidemic MSM-associated lineages (16). Information regarding pairwise SNP differences between the pairs was retrieved from the same study.

10.1128/mBio.00254-21.10 TABLE S6 Information for S. flexneri 2a, S. flexneri 3a, and S. sonnei isolates used to investigate accessory genome dynamics during persistent infection (First tab). Pairwise SNP distances were retrieved from Allen et al. (16). Pair classification (i.e., carriage or reinfection associated) were based on SNP distance and time intervals between serial-isolation of the pairs, established by the same study. Information on S. flexneri 3a isolates used to investigate chromosomal structural variation and rearrangement (second tab). ENA accession and NCBI genome accession numbers for Illumina and PacBio data are listed accordingly. Download Table S6, XLSX file, 0.02 MB. © Crown copyright 2021. 2021 Crown https://creativecommons.org/licenses/by/4.0/ This content is distributed under the terms of the Creative Commons Attribution 4.0 International license.

Each pair (*n *=* *57) was sampled from the same patient at two time points ranging from 1 to 1,862 days apart for S. flexneri and from 1 to 1,353 days apart for S. sonnei. As established previously (16), we defined a pair of isolates serially sampled from the same patient at time points ranging 6 to 176 days, with genetic distances ranging between 0 and 7 SNPs, as being associated with carriage, and pairs of isolates serially sampled between 34 and 2,636 days, with genetic distances of 10 to 1,462 SNPs, as being associated with reinfection (16). Here, we simplify these nomenclatures as “carriage-associated” and “reinfection-associated” isolate pairs. Using these definitions, the data set used for analysis comprised of 23 carriage-associated and 11 reinfection-associated pairs for S. flexneri and 15 carriage-associated and 8 reinfection-associated pairs of S. sonnei (see Table S1). All metadata for the individual isolates used in this study and the Sequence Read Archive (SRA) accession numbers are listed in Table S1 in the supplemental material.

### Extension study of *S. flexneri* 3a isolates, including long-read sequenced isolates.

Sixteen epidemic sublineage MSM-associated S. flexneri 3a isolates (3) were used to determine large structural variation and genome rearrangement of *Shigella* over time. These isolates were serially isolated from eight individuals between 9 and 911 days apart and were sequenced using both Illumina and PacBio technologies (Fig. 2A and B). For one patient (sampled at a 154-day interval), PacBio sequencing was only successful for the earlier isolate. Illumina sequencing for the isolates were generated at the Wellcome Trust Sanger Institute, as previously described (3). For this study, the 16 isolates were retrieved from the GBRU reference laboratory archives (where they are stored in darkness, at an ambient temperature, on Dorset’s egg medium), and DNA was extracted for long-read sequencing as previously described (11). DNA from each sample was sequenced on a Pacific Biosciences RS Sequel at the Centre for Genomics Research at the Institute for Integrative Biology, University of Liverpool.

To facilitate understanding, the isolates and nearly complete genome assemblies in this extension study have been abbreviated to meaningful titles to reflect the epidemiology and sequencing technology used. These names comprise the number of intervening days between serial isolations, the time point (A or B, i.e., the first and second time points, respectively), and sequencing technology (Illumina [I] or PacBio [P]) (Fig. 3B). For example, 20BP is the PacBio-sequenced genome of the second isolate taken from a patient whose isolates were sampled 20 days apart. The full key and genome accession numbers are provided in Table S2.

### Sequence processing and assembly.

Illumina sequencing data were adapter and quality trimmed using Trimmomatic v0.38 (53), and draft genomes were assembled using Unicycler v0.4.7 (54). PacBio data were assembled using Canu v1.6 (55) and iteratively polished using SMRT tool (Arrow) v6.0.0 (https://github.com/PacificBiosciences/GenomicConsensus). This generated genomes with a variable number of contigs (between 3 and 17). These draft genomes were reordered against the completed reference genome 20BP (described below) manually using a combination of pairwise all-by-all basic local alignment search tool (BLAST) and Bedtools v2.27.1 (56).

### Generation of a public isolate and complete genome of an internationally important pathogen.

For one PacBio-sequenced genome (20BP), three contiguous sequences were generated that corresponded to the bacterial chromosome, the virulence plasmid, and the pKSR100 resistance plasmid. To complete this genome for use as a downstream reference, circularization at *dnaA* was achieved manually by self-BLAST and removal of inverted repeat regions using Bedtools v2.27.1. 20BP was isolated from a dysenteric patient in the East of England who was male, aged 16 to 60 years old, and had no recorded travel history. The isolate belongs to sublineage A of an internationally disseminated MSM-associated lineage of *S. flexneri* 3a (3). Since this isolate was an internationally important pathogen, the cognate isolate has also been deposited at NCTC under accession number NCTC 14607.

### Pangenome and pairwise homologous sequence search.

All assembled draft genomes were annotated using Prokka v1.13.3 (57), and pangenome analyses were performed using Roary v3.12.0 (58), run without splitting paralogs. To determine the gain and loss of genes, pairwise homologous sequence searches were carried out using Roary between pairs serially isolated from individual patients at two time points. Accessory genes present in the first isolate and absent in the second were classified as loss, while genes absent in the first isolate and present in the second were classified as gain. To account for variations of gained or lost genes contributed by an *in silico* artifact (e.g., misassembly, misannotation), seven synthetic read sets with lengths of 36 to 90 bp and variable insert sizes (see Table S2) were generated from each of the complete genomes of S. flexneri 20BP and S. sonnei Ss046 (GenBank assembly accession GCA_000092525.1) using the randomreads.sh script from the BBMap package (59). These synthetic read sets were then assembled, annotated, and underwent pairwise comparisons (as described above). Comprehensive pairwise comparisons were carried out among the seven synthetic draft genomes generated from each reference genome. Each draft genome assembly from a particular read length was individually compared to the six genomes assembled at various lengths, generating a total of 42 pairwise comparisons for each species.

### Detection of previously characterized accessory genome elements.

The detection of genetic determinants conferring AMR was performed using AMRFinder v3.1.1b (60), and genes with >80% coverage and >90% nucleotide identity to reference sequences in the AMRFinder database were defined as present. The –organism option was applied and set to *Escherichia* to get an organism-specific result and screen for point mutations. The presence of pKSR100, pCERC1, and spA plasmids was inferred using short-read mapping with BWA mem v1 (61) against the pKSR100 from S. flexneri 20BP, pCERC1 from E. coli S1.2.T2R (GenBank accession JN012467), and spA from S. sonnei Ss046 (GenBank accession CP000641). Mapping of >80% sequence coverage across the reference with >90% nucleotide sequence identity was used to define plasmid presence. Contiguous sequences containing acquired AMR genes were also examined for plasmid replicons using PlasmidFinder v2.1 to search against the *Enterobacteriaceae* database from PlasmidFinder. To identify associations of AMR genes with known and/or related plasmid sequences and potential species origins, contiguous sequences containing AMR genetic determinants that were gained during carriage were also compared against the NCBI nonredundant database using MegaBlast. Phage elements in the 20BP reference genome were predicted using PHASTER (62).

### Determining pairwise SNP distances among *S. flexneri* 3a isolate pairs.

Genetic distances between each pair of isolates sequenced from the MSM-associated S. flexneri 3a epidemic sublineage were identified against the 20BP reference. First, SNPs were called by mapping sequence data against the chromosome and the associated virulence plasmid of the reference genome, as previously described (3). The short-read Illumina data were mapped directly, and the PacBio draft assemblies were shredded to simulated data of 100 bp in length with a 250-bp insert size every three bases along a circular chromosome, as previously described (11). Then, mapping was performed with smalt v0.7.6 (https://www.sanger.ac.uk/tool/smalt-0/), recombinant and invariant sites were removed using gubbins v2.3.4 (63), invariant sites were identified using snp-sites v2.4.1 (64), and pairwise distances were counted (number of nonmatching bases). To determine the SNP distance between each pair as a result of mapping the second to first isolate, short-read Illumina data were mapped against the PacBio genome of the earlier isolate using BWA mem (61), and variants were identified using BCFtools v1.9-80 (http://samtools.github.io/bcftools/).

### Structural rearrangements and functional annotation.

In order to detect structural variations and genome rearrangements among pairs, the Synteny and Rearrangement Identifier (SyRI) package was used. First, the 14 PacBio-assembled draft genomes were reordered against the complete reference genome of 20BP using chroder, part of the SyRI v1.3 software package (65). Then, reordered genomes were individually aligned against 20BP reference genome using NUCmer v3.1. Alignment coordinates generated were used as input for SyRI to detect structural variation between isolate pairs. The output of SyRI was compared between the first and second isolates from each pair. Common variants detected in both isolates of a serial pair indicated differences compared to the 20BP reference genome, whereas unique variants detected between the isolates from a pair indicated variation between the pair. Insertions and inversions detected by SyRI were evaluated by visualizing pairwise comparison of PacBio draft assemblies using Artemis Comparison Tools v18.1.0 (66). Mapping of short and long reads at regions of intra-isolate variation was performed to confirm duplications and deletions detected by SyRI and verified manually using Artemis v18.1.0 to visualise coverage at the variable region (67). Coordinates of the structural variants identified among the seven S. flexneri 3a pairs (according to the location of the 20BP reference genome) were parsed to Circos v0.69-9 for visualization (68).

To explore the functional features of the structurally variable genomic regions, the locations of the regions were identified along the 20BP chromosome, and genome sequences were manually checked for IS elements, as identified using ISEScan v1.7.2 (69). Functional assignment of the GO category for genes in the 20BP reference chromosome was predicted using RAST v2.0 (70), which annotates CDS by comparison to the curated FIGfams protein family database (71) and assigns genes into different functional categories.

### Statistical analyses.

All statistical analyses were performed using R v3.6.1. Statistical differences between accessory gene content variation among isolate pair classification groups (i.e., carriage versus reinfection and data versus control) were tested using the Mann-Whitney U test (72) with the wilcox.text() function. Linear regression analysis of SNP distance against gene content variation among isolate pairs was performed with the lm() function. Correlation between gene content variation and SNP distance was tested using the Spearman’s rank correlation coefficient with the cor.test() function. Statistical difference in the proportion of genes in each GO category was tested using chi-square tests with the chisq.test() function, using the raw values.

### Data availability.

PacBio data have been deposited in the European Nucleotide Archive under study accession number PRJEB39785, and the accession numbers for each isolate are listed in Table S6.
