# Supplementary figures and images for: Accessory Genome Dynamics and Structural Variation of Shigella from Persistent Infections
Source: mBio. 2021 Apr 27;12(2):e00254-21. doi: 10.1128/mBio.00254-21 (PMC8092226; doi:10.1128/mBio.00254-21)

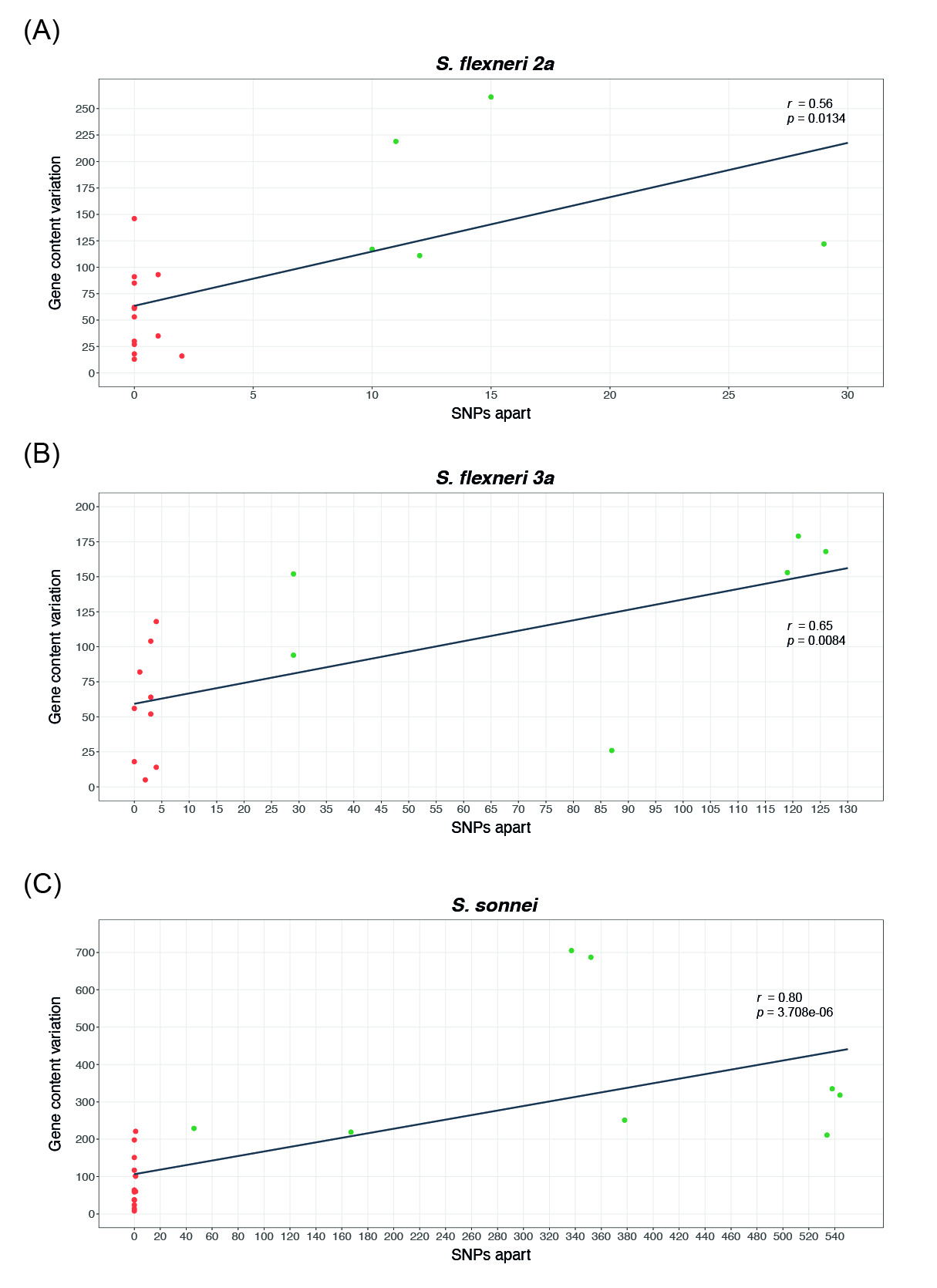

Supplement: FIG S1 [file mBio.00254-21-sf001.tif]

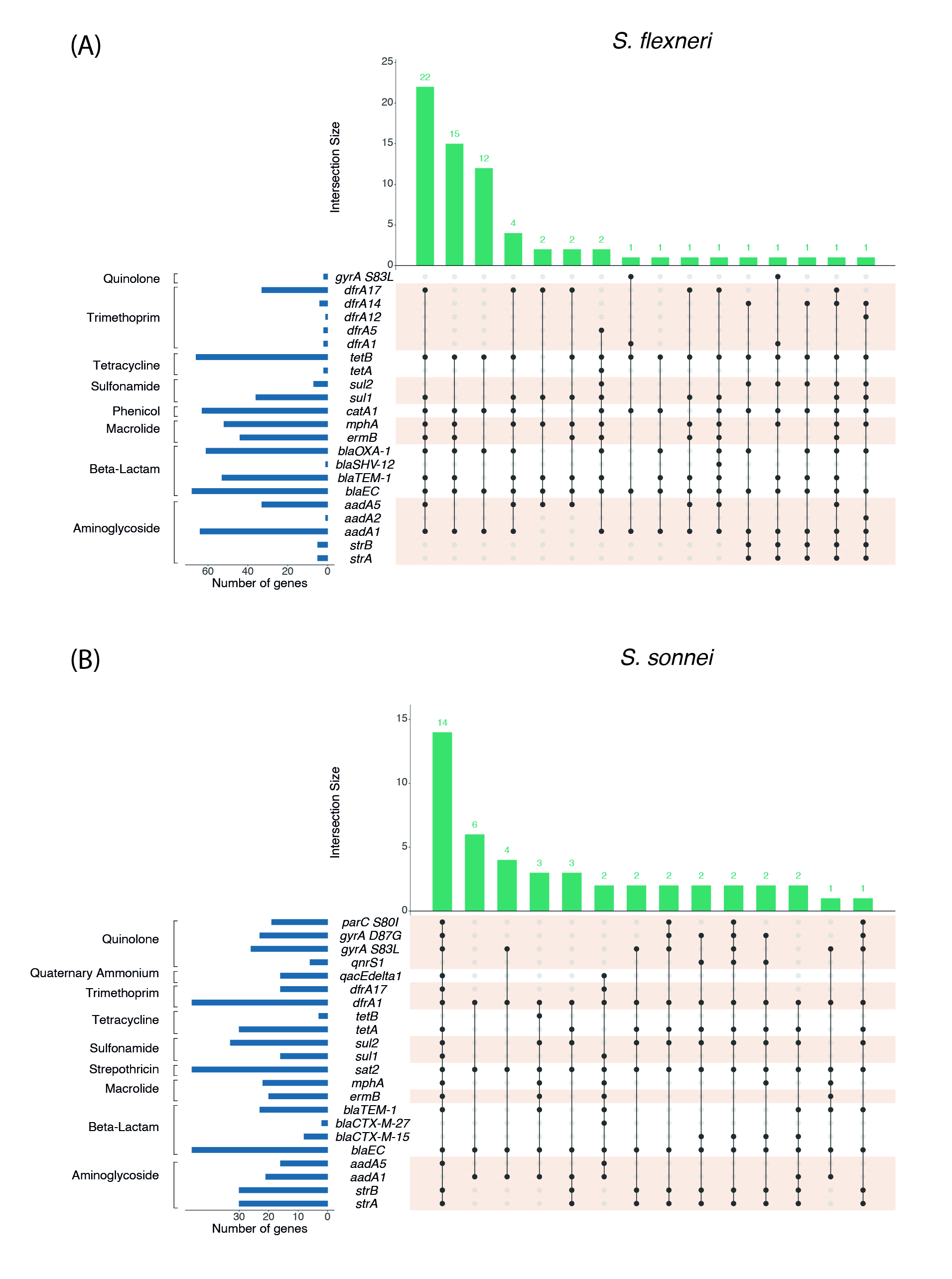

Supplement: FIG S2 [file mBio.00254-21-sf002.tif]

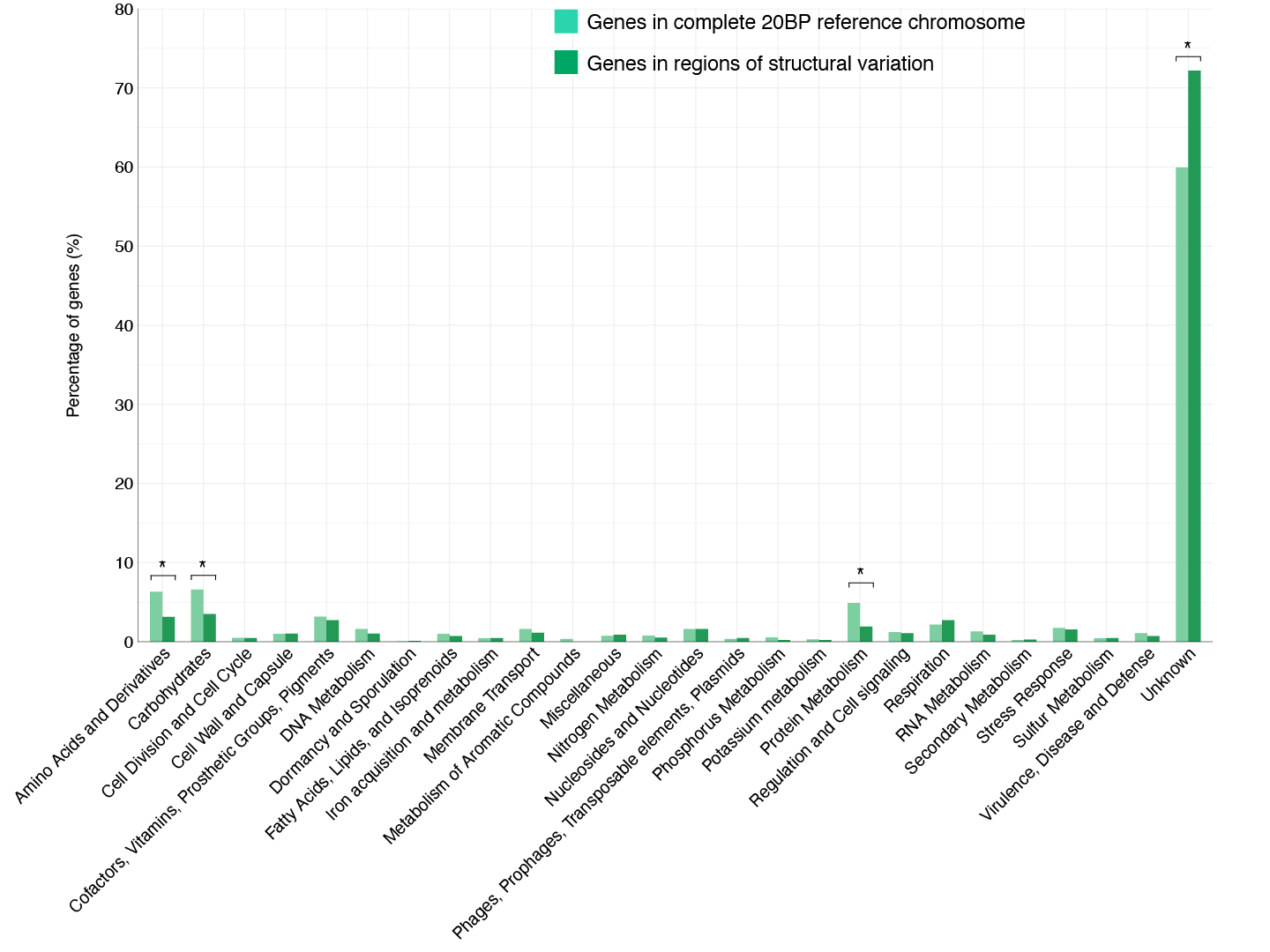

Supplement: FIG S4 [file mBio.00254-21-sf004.tif]

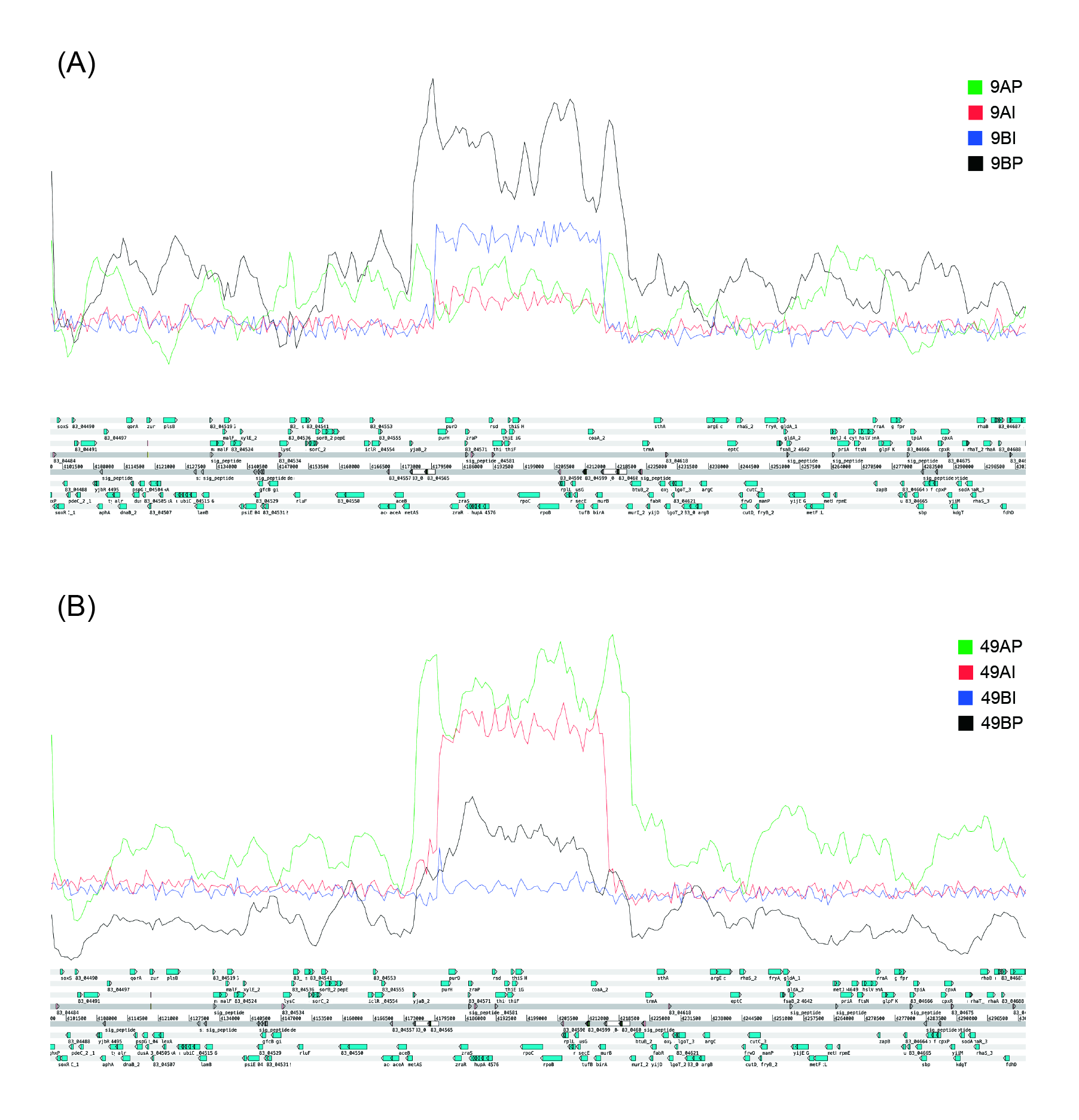

Supplement: FIG S3 [file mBio.00254-21-sf003.tif]
