# Supplementary material for: Accessory Genome Dynamics and Structural Variation of Shigella from Persistent Infections
Source: mBio. 2021 Apr 27;12(2):e00254-21. doi: 10.1128/mBio.00254-21 (PMC8092226; doi:10.1128/mBio.00254-21)
Supplement: TABLE S1 [file mBio.00254-21-st001.docx]

| **Serotype** | **Carriage associated pairs** | **Reinfection associated pairs** | **Total pairs** |
| --- | --- | --- | --- |
| *S. flexneri* 2a | 14 | 5 | 19 |
| *S. flexneri* 3a | 9 | 6 | 15 |
| *S. sonnei* | 15 | 8 | 23 |
| **Total** | **38** | **19** | **57** |

**Table S1**. Number of isolate pairs analysed in the current study, broken down by *Shigella* subtypes and classification as carriage associated or reinfection associated.
