# Supplementary material for: Accessory Genome Dynamics and Structural Variation of Shigella from Persistent Infections
Source: mBio. 2021 Apr 27;12(2):e00254-21. doi: 10.1128/mBio.00254-21 (PMC8092226; doi:10.1128/mBio.00254-21)
Supplement: TABLE S2 [file mBio.00254-21-st002.docx]

| **Reference genome** | **Read length (bp)** | **Insert size** | **CDS number** |
| --- | --- | --- | --- |
| S. flexneri 20BP (GCA_904066025) | 36 - 100 | 22-428 | 4215 |
|  | 40 - 100 | 30-420 | 4217 |
|  | 50 - 80 | 90-360 | 4215 |
|  | 60 - 90 | 90-360 | 4215 |
|  | 70 - 100 | 90-360 | 4234 |
|  | 80 - 100 | 110-340 | 4230 |
|  | 90 - 100 | 130-320 | 4230 |
| S. sonnei (GCA_000092525.1) | 36 - 100 | 22-428 | 4230 |
|  | 40 - 100 | 30-420 | 4228 |
|  | 50 - 80 | 90-360 | 4233 |
|  | 60 - 90 | 90-360 | 4232 |
|  | 70 - 100 | 90-360 | 4238 |
|  | 80 - 100 | 110-340 | 4238 |
|  | 90 - 100 | 130-320 | 4247 |

**Table S2.** Number of CDS annotated from draft genome assemblies generated from synthetic reads of various length and insert size of 20BP and Ss046 complete reference genomes.
